# Message from the Editor

**Published:** 2009-10

**Authors:** Pramila Bajaj

**Figure:**
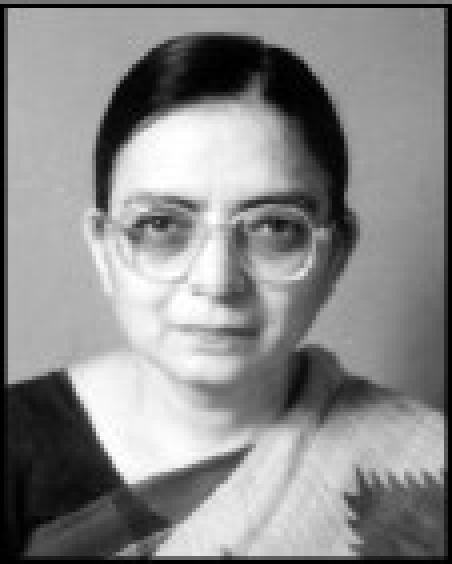
Pramila Bajaj Editor, IJA

Dear Members,

With a deep sense of gratitude, I express my heartfelt thanks to all members for reposing faith in my capabilities and given responsibility as the editor from Jan 07 till Dec 09 for our esteemed journal.

After completing the golden jubilee of the volume published, IJA had marched on to publications of its 51^st^ volume, in 2007. It was my immence pleasure and privilege to lead our pride journal as a first lady editor. During my three years tenure, I strived hard to turn this esteemed journal into something which we all can truly be proud of.

Dear members, Editor office started working from Feb 07 onward at 25, Polo Ground, Udaipur. The office was well equipped with furniture, computer and printer. To start with IJA account in 2007, an account in SBI was opened with Rs. 1000/-deposited by myself. Later on Rs. 2 lacs was deposited by secretary, ISA on 27.6.07. At the time of printing of June 07 issue of IJA and onwards some amount was paid by Editor office (Amount collected from advertisers and subscribers) and by ISA secretary.

During first year of Editorship (2007), in the first governing council meeting held at Jodhpur the rate of printing (same as it was before), change of color of covering page and to print contents of articles on first page was appreciated and approved.

In the second year of Editorship, proposal for publishing of 7 issues of IJA was agreed, which includes 6 regular issues of IJA and one P.G.issue of IJA as supplement issue. This announcement was published in June 08 & Aug 08 issues of IJA that is:

Editor office will publish Six regular issues of “Indian Journal of Anaesthesia” every year from year 2008 onwards. “Post Graduate issue” will be published as a supplementary issue in the month of October.

The first supplementary issue(P.G) of 132 pages was printed and posted to members of ISA.

The proposal of publishing of seven issues of IJA and its budget was approved by General Body meeting held at Jodhpur December 2008.

## The budget of Editor office always remains on following headings:

**Table:** 

1. Printing per issue(130 pages)	441022(9100 copies)	
2. Postage per issue(130 pages)	103285(9100 copies)	
3. Annual Expenditure:	
a.Computer operator salary per year:	60000
b.Computer maintenance per year:	5000
c. Telephone expensesper year:	10000
d. Miscellaneous per year:	5000

This does not include expenditure of well equipped Editor office except payment of Internet connections.

In June 07 no. of copies of IJA printed were 6700(100 pages).

In Aug 2009 no. of copies of IJA printed were: 9100(130 pages).

During the tenure of III year of editorship(2009) in first and II Governing Council meeting held at Lucknow and Chennai respectively, the proposal for publishing 7 issues was withdrawn which was approved by G.B.meeting held at Jodhpur 2008. It was further instructed that the issues that will come out from now should not have more than 80 pages, each as compared to previous issue of 130 pages during last two & half years upto Aug 2009. It was further instructed to publish 100 pages of P.G issue in Oct 2009.

Many of the articles which were under final selection phase and acceptance was already sent to them, and were planned to be published in forthcoming issues, but with decision of reduction of number of pages and omission of Oct 09 regular issue, this is not possible to accommodate them in forthcoming issue of IJA.

Defaulter member who have deposited Rs. 1000/- to secretary ISA for regular supply of IJA, Editor office posted them, back issue of available IJA.

The survival and growth of a scientific journal depends on how the journal conducts reviews before publication, how national and international author perceive the quality of the journal for presenting their articles, how the journal becomes a forum for discussion on important technical issues, and how fast results of scientific research are accepted, analysed and reported. About the content of a published article, I would like to make it loud and clear that a stringent system was worked out and every article was sent to a reviewer who is an expert in the field. The identity of the author and institution were blanked out so that the reviewer has no bias and then a published article though peer reviewed is still open for comments which generates interest on the topic and it is another way of auditing our own work.

During the tenure of my editorship each articles received from different corner is being peer reviewed by two or three peer reviewed and finally by editorial team. The final PDF file was checked by author as well as by editorial team. All the process of reviewed articles, sending for peer reviewer was done by email services only.

The progress of a scientific journal will eventually be determined by its scientific content; no medical journal can survive without it. The success of author and journal is interdependent. I am extremely thankful to the editorial board members and peer reviewers for their constant encouragement and helped me in progress of journal.

I worked hard and journal has prospered and earned credibility amongst all the reader members.

Pramila Bajaj

Editor, IJA

